# Next-generation T cell immunotherapy: overcoming exhaustion, senescence, and suppression

**DOI:** 10.3389/fimmu.2025.1662145

**Published:** 2025-10-13

**Authors:** Guangmei Li, Dengju Li, Xiaojian Zhu

**Affiliations:** Department of Hematology, Tongji Hospital, Tongji Medical College, Huazhong University of Science and Technology, Wuhan, Hubei, China

**Keywords:** T-cell enhancement, tumor immunotherapy, T-cell exhaustion and aging, metabolic regulation, microenvironment optimization

## Abstract

T-cells are a core component of tumor immunotherapy because of their potent ability to identify and kill cancer cells. Yet efficacy is limited by exhaustion, senescence, metabolic dysregulation, an immunosuppressive tumor microenvironment (TME), and limited persistence. This review analyzed these key issues and proposed targeted improvement strategies. Emerging approaches encompass pharmacological modulation of T cell activation and survival pathways, epigenetic reprogramming to reverse exhaustion and senescence, metabolic engineering, combinatorial targeting of immunosuppressive TME components and advanced genetic tools, notably CRISPR-Cas9–based CAR-T optimization, which exemplifies how precise genome editing can enhance therapeutic efficacy. We review the progress and prospects of T-cell improvement strategies in tumor immunotherapy, emphasizing the need for further exploration to enhance the broader application and long-term efficacy of T-cell therapies. This review highlights recent advances and future directions in T-cell engineering, metabolic modulation, and microenvironment targeting, aiming to translate innovations into effective cancer immunotherapies.

## 1 Background

Tumor immunotherapy leverages the immune system to identify and destroy cancer cells and offers a more targeted approach with fewer side effects, while chemotherapy and radiotherapy often harm healthy tissues and cause more side effects. Since the late 19th century, when William Coley first used bacterial toxins to induce an immune response to treat cancer (1), tumor immunotherapy has advanced significantly, highlighted by FDA approval of immune checkpoint inhibitors like ipilimumab in 2011 (2), greatly improving patient survival and durable immune responses against cancer (3).

In many tumor immunotherapies, T cells are the core of the adaptive immune response due to their strong adaptability and precision in targeting tumor cells. In contrast, NK-cell–based therapies often exhibit limited persistence and fail to generate antigen-specific memory. Similarly, macrophage-based approaches are highly plastic and can be reprogrammed into pro-tumor phenotypes within the tumor microenvironment. T cells uniquely combine durable survival, antigen-specific recognition, and engineering flexibility, underscoring their central role in immunotherapy development. By targeting tumor-associated antigens presented by major histocompatibility complex (MHC) molecules, cytotoxic T cells (CTL) can specifically identify and destroy cancer cells without damaging normal tissues. This highly specific targeting provides T cells with distinct advantages over conventional cancer therapies.

Despite the remarkable success of T cell–based immunotherapies, substantial challenges remain in achieving durable and universal responses across diverse cancer types. Tumor cells are highly heterogeneous and often express a spectrum of immunosuppressive ligands, thereby enabling immune escape through multiple mechanisms within the tumor microenvironment. Recent T cell-based immunotherapies, including immune checkpoint inhibitors, chimeric antigen receptor (CAR)-T cell therapy, TCR-engineered T cells, bispecific T-cell engagers, and tumor-infiltrating lymphocyte (TIL) therapy, have expanded the therapeutic landscape. These approaches restore T-cell activity, block inhibitory signaling, and induce sustained antitumor responses, with CAR-T therapy showing particularly notable efficacy in hematologic malignancies. Moreover, emerging studies have highlighted the key role of T-cells in adoptive cell therapy. It is crucial to optimize T-cell-based immunotherapy, including T-cell activity, proliferative capacity, persistence, and cytotoxicity. In the future, innovative strategies to improve T-cell function are expected to significantly enhance the clinical effectiveness of immunotherapy. This review summarizes the strategies for improving T cell function and explores their potential in addressing tumor immune escape and enhancing therapeutic efficacy.

## 2 Mechanisms of T-cell activation and functional regulation

In immunotherapy, antigen recognition by T cells and the associated signaling pathways involve a complex network of molecular mechanisms. T cells can specifically identify and respond to foreign pathogens or abnormal cells while avoiding attacks on the body’s tissues. By modulating these mechanisms, the antitumor and antiviral capabilities of T cells can be enhanced. A deeper understanding of these processes may offer new avenues for optimizing T-cell function in immunotherapy, ultimately improving treatment outcomes.

### 2.1 Antigen presentation

Antigen-presenting cells (APCs) capture antigens via phagocytosis or receptor-mediated endocytosis, degrade them into peptides, and bind these peptides to major histocompatibility complex (MHC) molecules to form MHC-antigen complexes. These complexes then interact with T-cell receptors (TCRs) to deliver an initial activation signal, which is fundamental for T cells to recognize specific antigens and initiate responses. APCs deliver a second signal via co-stimulatory molecules (e.g., CD80/CD86 interacting with CD28 on T cells) to ensure the complete activation of T cells and prevent them from entering a hypofunctional or tolerant state. APCs also secrete cytokines, to deliver a third signal. These cytokines regulate the growth and differentiation of T cells, guiding their differentiation into effector and memory T cells. APCs play a key role in the initiation of T cell immune responses by determining the activation, expansion, and functional status of T cells.

### 2.2 Mechanisms of MHC class I and II antigen presentation

MHC class I molecules are primarily responsible for presenting endogenous antigens to CD8 + T cells (4). In the endoplasmic reticulum (ER), unfolded MHC I molecules bind to calnexins to ensure proper folding. Subsequently, MHC I binds non-covalently to β 2-microglobulin to stabilize the structure. The transporter associated with antigen processing (TAP) transports the peptide to the endoplasmic reticulum for assembly into the antigen-binding groove of MHC I. This complex is then transported to the cell surface via the Golgi apparatus. CD8 + T cells are activated after their receptors bind to antigenic peptides on MHC I, leading to the release of perforin and granzyme, which directly kill infected or cancerous cells.

MHC class II molecules primarily present exogenous antigens to CD4 + helper cells (5). MHC class II molecules are synthesized in the ER and bind to invariant chains to prevent endogenous antigens from occupying the antigen-binding groove. HLA-DM then removes Class II-associated invariant chain peptide (CLIP), allowing exogenous antigen peptides to bind to MHC II. The MHC II-antigen complex is transported to the cell surface, where it interacts with the CD4 + T cell receptor to activate CD4 + T cells, leading to the activation of CD8 + cytotoxic T and B cells, coordinating the immune response (6).

Tumors achieve immune evasion through dysregulation of the MHC expression pathway. Therapies that use IFN-γ to restore MHC I expression can reenable CD8+ T cells to recognize tumors. Additionally, enhancing the expression of MHC II on antigen-presenting cells (APCs) can enhance the helper function of CD4+ T cells, which is crucial for maintaining an effective anti-tumor immune response and improving the success rate of vaccines and immune checkpoint blockade therapies.

### 2.3 TCR signaling

#### 2.3.1 Structures and signaling pathways

TCR signaling cascade starts with antigen recognition, leading to the recruitment of the Src family kinase, Lck, to the TCR complex. Lck phosphorylates immunoreceptor tyrosine-based activation motifs (ITAMs) on CD3 subunits, a process that subsequently recruits and activates the protein tyrosine kinase zeta-chain–associated protein kinase of 70 kDa (ZAP-70) (7). Once activated, ZAP-70 phosphorylates the key adaptor proteins LAT and SLP-76 (7), which are essential for assembling signaling complexes. Phosphorylated LAT acts as a docking site for several proteins, including PLCγ, GRB2, and Gads (7), which initiates downstream signaling. The activation of PLCγ is crucial for the calcium signaling pathway. This signaling cascade enhances T-cell activity and triggers cellular responses.

#### 2.3.2 Costimulatory signals and their effects

**Figure 1:**
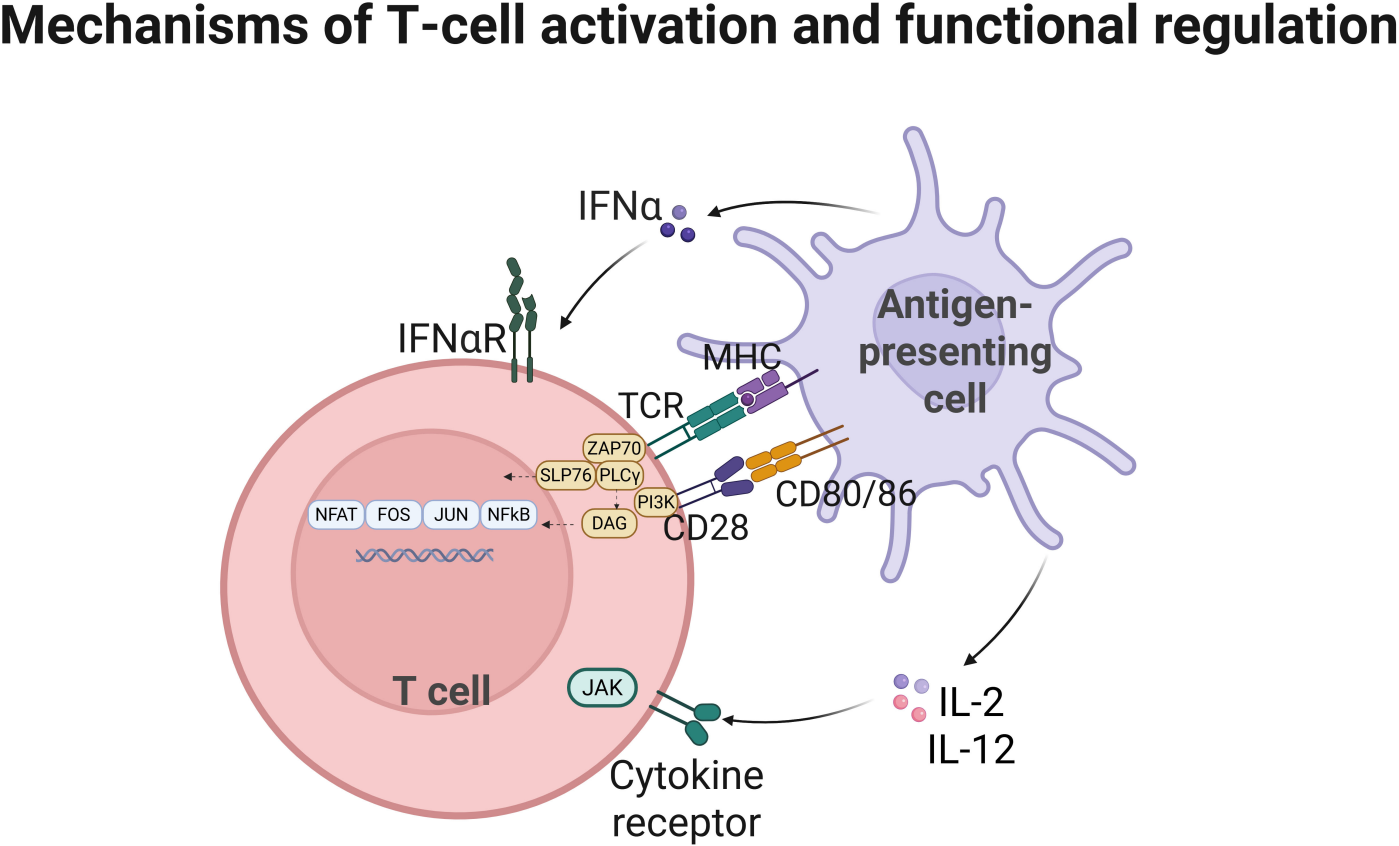
Mechanisms of T-cell activation and functional regulation T cells recognize antigens via T-cell receptors (TCR) presented by antigen-presenting cells (APCs) through MHC molecules, with co-stimulatory signals and cytokines promoting T cell activation, proliferation, and differentiation into effector and memory cells.

While recognition of peptide–MHC complexes by TCRs provides the initial activation signal, this alone is insufficient for full T-cell activation. A crucial second signal is delivered through co-stimulatory pathways, most prominently the interaction of CD28 on T cells with CD80 (B7.1) or CD86 (B7.2) on APCs (8). This engagement promotes T-cell proliferation, cytokine secretion, survival, and effector functions. These co-stimulatory signals activate downstream pathways such as PI3K–AKT and ERK/MAPK, reinforcing T-cell activation (**Figure 1**).

The design of CAR-T cells is a direct clinical application of these TCR signaling principles. CARs incorporate ITAM-containing CD3ζ domains to initiate activation and costimulatory domains (e.g., CD28, 4-1BB) to enhance T cell persistence and function. This synthetic biology approach exemplifies how targeted manipulation of T cell signaling pathways can generate potent antitumor immunity. **Table 1** presents the mechanisms of T cell activation.

**Table 1:** Mechanisms of T cell activation.

Signal Type	Key Molecules	Primary Function and Effect	Notes
First Signal (Antigen Recognition)	TCR recognition of pMHC complex	Provides antigen-specific recognition; serves as the foundational and initiating signal for T cell activation.	TCR: T cell receptor, responsible for specific antigen recognition. pMHC: Peptide-Major Histocompatibility Complex, presented by Antigen-Presenting Cells (APCs).
Signal Initiation	Lck phosphorylates ITAMs	Initiates downstream signal transduction cascades.	Lck: A Src-family tyrosine kinase, responsible for initiating phosphorylation. ITAMs: Immunoreceptor Tyrosine-based Activation Motifs, which are signaling modules on CD3 molecules.
Signal Transduction	ZAP-70 is recruited and activated	Relays and amplifies the upstream signal for propagation to downstream effectors.	ZAP-70: A tyrosine kinase that binds phosphorylated ITAMs; a critical node in signal transduction.
Signal Assembly	ZAP-70 phosphorylates LAT and SLP-76	Forms a signalosome, serving as a scaffold platform for the assembly of multiple downstream signaling pathways.	LAT: Linker for Activation of T cells, a crucial adaptor protein. SLP-76: Another key adaptor protein that functions cooperatively with LAT.
Downstream Pathway​​	PLCγ is recruited and activated	Hydrolyzes PIP_2_, initiating the calcium signaling pathway and triggering cellular responses.	PLC: Phospholipase C gamma, acts as a bridge connecting upstream signals to downstream pathways such as calcium flux. Calcium signaling pathway: A rise in intracellular calcium concentration is a pivotal event for T cell activation.
Second Signal (Co-stimulation)	CD28 (on T cell) binding to B7 (CD80/CD86 on APC)	Provides a critical co-stimulatory signal to ensure full activation, prevent anergy, and promote proliferation, survival, and cytokine production.	B7: A family of co-stimulatory molecules on APCs, including B7.1 (CD80) and B7.2 (CD86).
Downstream Pathway	Activation of PI3K-AKT and ERK/MAPK pathways	Activated by the co-stimulatory signal; collectively promote T cell activation.	
Third Signal (Cytokines)	Cytokines secreted by APCs	Regulate T cell growth and differentiation, directing their development into effector and memory cells.	

The corresponding immunotherapy strategies directly target these signals: dendritic cell vaccines aim to optimize antigen presentation and co-stimulation. Cytokine therapy provides strong cytokine signals to drive T cell expansion and memory differentiation. Both are clinical transformation examples targeting the fundamental mechanism of the APC-T cell interaction.

## 3 Problem analysis

Before discussing targeted solutions, it is essential to clarify the major challenges faced by immunotherapy within the tumor microenvironment. Such an analysis provides the conceptual foundation for the problem-oriented strategies outlined in the following section.

### 3.1 Functional exhaustion of T-cells

T cell failure is a common dysfunction in chronic infections and cancer. This process not only impairs T cell immune responses but also involves metabolic alterations and unique transcriptional programs that distinguish exhausted effector T cells (TEF) from memory T cells (TMEM) (9). Exhausted T cells exhibit high expression of inhibitory receptors like PD-1, Lag-3, and Tim-3, further suppressing their function. Blocking PD-1 can reverse T cell migration arrest and partially restore effector function (10). These findings underscore PD-1 as a nexus integrating T cell receptor signaling, metabolic fitness, and epigenetic programming—a complexity that demands combinatorial targeting rather than monotherapy. Targeting this exhaustion axis—driven by chronic antigen exposure and manifesting as proliferative arrest, cytotoxic collapse, and immunosuppressive checkpoint upregulation—provides a strategic entry point for combinatorial therapies to reinvigorate T cell function.

### 3.2 T cell aging mechanisms

Senescent T cells display distinct surface markers, including high expression of senescence-associated β-galactosidase (SA-β-galactosidase), reduced expression of costimulatory molecules CD27 and CD28, and upregulation of inhibitory receptors such as Tim-3. Changes in these markers and their functional features often lead to T-cell dysfunction, which affects their antitumor capacity (11). For instance, loss of CD28 reduces the ability of T cells to receive costimulatory signals critical for activation, while increased Tim-3 expression correlates with reduced cytokine production and survival. Importantly, such surface markers also provide potential therapeutic entry points—for example, blocking inhibitory receptors or rejuvenating senescent T cells through telomerase activation or metabolic reprogramming. Thus, integrating the functional and therapeutic implications of aging markers is essential for improving immunotherapeutic efficacy. Understanding the mechanisms of aging and exploring effective reversal or intervention strategies are expected to improve the success rate of immunotherapy and prognosis of cancer patients.

### 3.3 Metabolic competition and inhibition in tumor microenvironment

Upon activation, T cells undergo profound metabolic remodeling. Effector T cells rely heavily on aerobic glycolysis to support rapid proliferation and effector function, whereas memory T cells depend primarily on fatty acid oxidation (FAO) and oxidative phosphorylation for long-term survival and surveillance (12). Specific enzymes such as hexokinase 2 (HK2) and pyruvate dehydrogenase (PDH) regulate glycolytic flux, while transporters such as glucose transporter 1 (GLUT1) and monocarboxylate transporter 4 (MCT4) mediate nutrient uptake and lactate export, respectively. Dysregulation of these pathways compromises T-cell survival and function. In the tumor microenvironment (TME), metabolic competition is intense: tumor cells with high glycolytic and amino acid demands deplete glucose, glutamine, and fatty acids, leaving T cells metabolically starved. Elevated potassium levels and inhibitory signals (e.g., PD-L1/PD-1 axis) further suppress T-cell metabolic fitness, blunting cytokine production and cytotoxicity (13). Clinically, therapeutic strategies targeting metabolic checkpoints—such as enhancing FAO in memory T cells or inhibiting tumor glycolysis—represent promising avenues for improving T-cell–based immunotherapies.

### 3.4 Tumor microenvironment-mediated suppression

The tumor microenvironment (TME) harbors multiple immunosuppressive mechanisms that impede effective T-cell–mediated antitumor responses (13). For example, in pancreatic cancer and ovarian cancer, dense stromal fibrosis forms a physical “immune barrier” that restricts T-cell infiltration (14, 15). In melanoma, tumor-associated fibroblasts (CAFs) secrete CXCL12, repelling T cells away from tumor nests (16). Meanwhile, myeloid-derived suppressor cells (MDSCs) accumulate in colorectal and lung cancers, releasing arginase and reactive oxygen species that suppress T-cell effector function (17). Moreover, vascular endothelial growth factor (VEGF) and other factors indirectly inhibit T-cell infiltration by altering blood vessel function (13). Together, these TME-derived factors synergistically dampen antitumor immunity and reduce the efficacy of immunotherapies. Consequently, rationally designed combination strategies that simultaneously target TME barriers (e.g., anti-VEGF therapy, CAF inhibition) and enhance T-cell resilience hold strong potential for improving clinical outcomes.

### 3.5 Limited persistence of T cells

**Figure 2:**
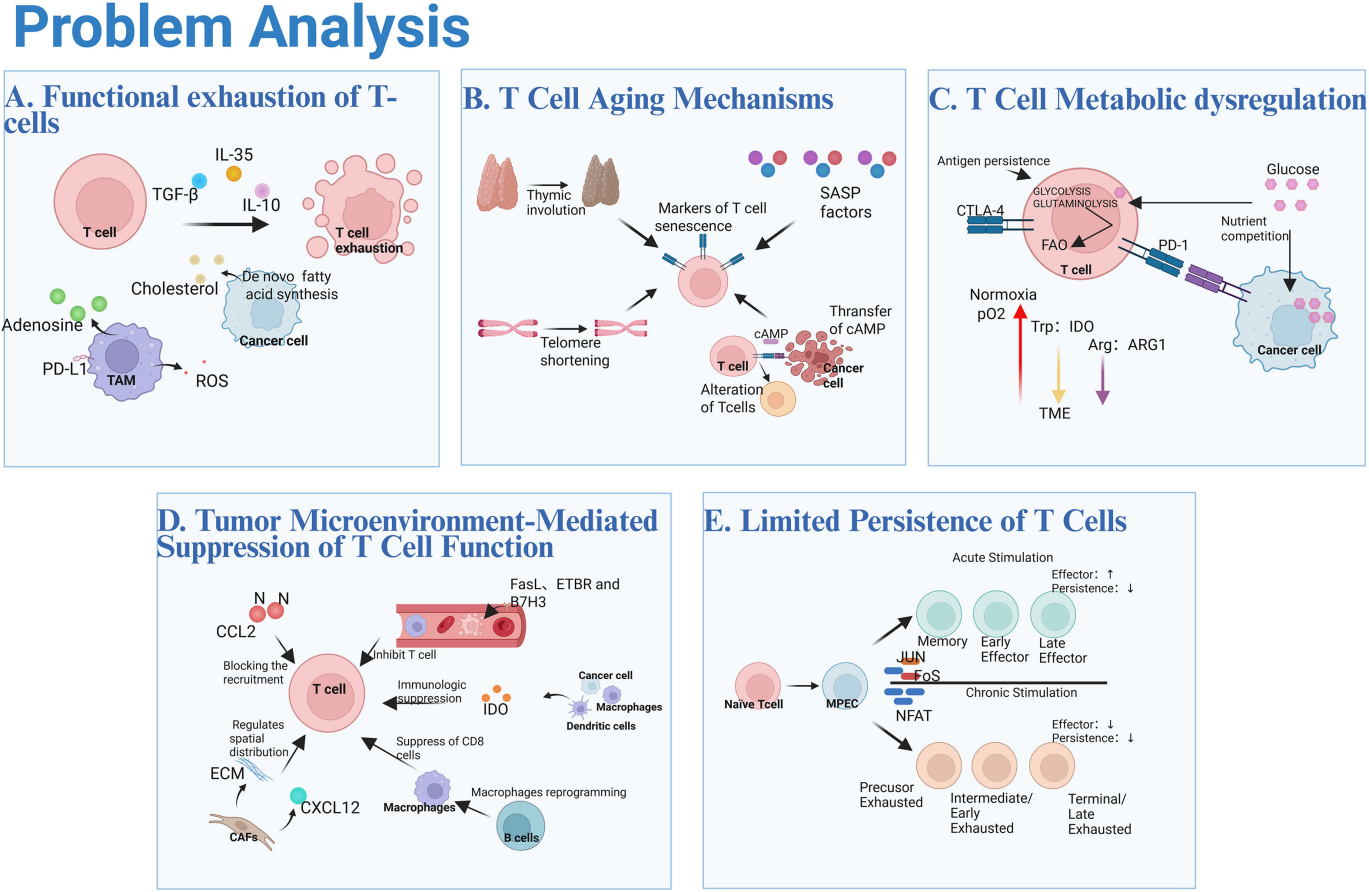
Problem analysis. **(A)** The accumulation of immunosuppressive cytokines is a key driver of T cell functional exhaustion. Tumor-associated macrophages (TAMs) exacerbate this process by secreting ROS, inducing PD-L1 expression and participating in adenosine metabolism, thus forming multiple immunosuppressive mechanisms. Lipid accumulation within tumor-infiltrating lymphocytes (TILs) induces metabolic stress and further impairs T cell function. **(B)** Thymic involution leads to a reduction in T cells, aging-related signals induce the senescence-associated secretory phenotype (SASP), and telomere shortening, along with changes in T cell phenotype and differentiation states, collectively drive T cell senescence. Moreover, Treg cells and tumor cells utilize cAMP to induce senescence in naïve and effector T cells, resulting in the loss of CD27 and CD28, thereby amplifying immunosuppressive effects in the tumor microenvironment. Markers of T cell senescence include KLRG1, CD57, and the recently identified receptor TIGIT. **(C)** Persistent antigen stimulation and inhibitory receptors like PD-1 and CTLA-4 remodel T cell metabolism, suppressing glucose and glutamine metabolism, impairing mitochondrial function, and increasing reliance on fatty acid oxidation. The tumor microenvironment worsens metabolic dysfunction with hypoxia, altered tryptophan and arginine metabolism, elevated lactate, and competition with cancer cells for glucose. **(D)** CCL2 indirectly inhibits the recruitment of T cells by recruiting MDSCs and macrophages. Tumor endothelial cells suppress T cell infiltration via FasL, ETBR, and B7H3. TME metabolic abnormalities, TAMs modulated by B cells, and CAFs via ECM capture and CXCL12-driven exclusion collectively impair T cell proliferation, infiltration, and antitumor activity while activating immunosuppressive cells. **(E)** Acute stimulation generates functional T cells, while chronic stimulation induces exhaustion. Both weaken CD8+ T cell persistence, reducing their long-term antitumor immunity efficacy.

T cell persistence is closely associated with the success of immunotherapy. Although effector memory T cells (TEM) can quickly respond to antigens, they lack persistence and rapidly decline after antigen clearance, leading to insufficient long-term immunity. There are multiple mechanisms for the poor persistence of T cells, including telomere shortening which leads to accelerated replicative senescence (18). Cytokine dependence, especially the insufficient signals of IL-7 and IL-15, limits long-term survival (19). Epigenetic regulation, including changes in DNMT3A and TET2, affects memory differentiation and persistence (20). Metabolic stress and oxidative damage can damage the health of mitochondria, further shortening lifespan (21). Additionally, some current vaccines induce short-term responses dominated by TEM, which may result in inadequate long-term immune protection (22). In chronic infections, prolonged antigen exposure causes T cells to undergo functional decline (exhaustion) and replicative aging, leading to decreased function and impaired persistence (23–25). T-cell exhaustion further aggravates the failure of immune function; therefore, the main focus of future immunotherapy and vaccine design should be on converting exhausted T cells into durable stem-like memory subsets, aiming to achieve sustained tumor control through enhanced T cell persistence.(**Figure 2**).

Importantly, these mechanisms are not independent. T-cell aging accelerates the development of exhaustion, while metabolic stress in the tumor microenvironment further amplifies both processes by impairing mitochondrial function and nutrient utilization. In turn, these converging pressures critically undermine T-cell persistence, highlighting that therapeutic strategies must simultaneously address senescence, exhaustion, metabolic fitness, and TME-mediated suppression to achieve durable antitumor immunity.

## 4 Problem-oriented strategy

Having identified the key obstacles in immunotherapy, this section turns to problem-oriented strategies. We focus on metabolic reprogramming, checkpoint blockade combinations, targeting of suppressive cell populations, and microenvironmental remodeling. Collectively, these strategies aim to mitigate the limitations described in Section 3, enhance T-cell persistence and effector function, and facilitate the clinical translation of immunotherapeutic approaches.

### 4.1 Improve T cell activation and persistence

#### 4.1.1 Transformation of the antigen presentation process

Targeted optimization can be achieved by modifying the antigen presentation process, thereby addressing the issue of limited T cell persistence. MAPK inhibitors effectively suppress the proliferation of cancer cells and enhance the transcription of MHC-I genes, promoting the activation of CD8+ T cells and increasing their infiltration into solid tumors (26). Additionally, researchers have engineered dynamic lipid bilayers that co-present TCR and co-stimulatory signals in a physiologically ordered manner, sustaining T-cell expansion and cytokine (e.g., IL-2) support while avoiding the abrupt over-activation linked to single high-dose stimulation (27).

Despite their therapeutic potential, MAPK pathway inhibitors (such as BRAF/MEK inhibitors) are associated with hepatotoxicity (28), and in clinical practice adaptive or acquired resistance frequently emerges. Meanwhile, artificial cell systems or carriers based on lipid bilayers continue to face engineering and translational challenges in terms of large-scale manufacturing and *in vivo* delivery (29). Therefore, at the current stage, these strategies are more appropriately considered as adjuncts or components of combination therapies rather than standalone treatments, unless substantive breakthroughs are achieved in safety and delivery technologies.

#### 4.1.2 Regulation of T cell differentiation

Optimizing T cell activation signals, enhancing their differentiation status, and regulating their resistance to exhaustion are key research directions in the future. Dual costimulation (4-1BB/ICOS) amplifies the persistence of CD8+ T cells through the interaction of NF-κB/NFATc1 (30), significantly enhancing the efficacy of adoptive cell therapy and helping to prolong the disease-free survival of patients and reduce the risk of recurrence (25, 31).

The regulation of T-cell differentiation and an increase in the proportion of memory T-cells can enhance T-cell persistence. Tcf1 + PD-1 + T cells exhibit characteristics similar to those of stem cells (25, 31). By self-renewal, they continuously produce Tcf1− cells to participate in the killing of tumor cells, thereby supporting a long-term anti-tumor response. The regulation of the NR4A family receptors (NR4A1, NR4A2, NR4A3) can effectively enhance T cell activation and persistence by regulating the early activation state and promoting the differentiation of memory T cells (32). Moreover, epigenetic regulation via the inhibition of BCL-6 and BLIMP-1, key regulators of T follicular helper cells, can prolong the survival of CAR T cells (33). CD4 + CAR T cells not only show strong cytotoxic activity but also show less sensitivity to activation-induced cell death and express fewer inhibitory immune checkpoint receptors than CD8 + CAR T cells, resulting in greater persistence and antitumor activity (34, 35). This finding may enhance therapeutic effects by regulating the differentiation of CD4 + T cells. Furthermore, regulation of T cell polarization, particularly toward Th2 or Th9 cells, can significantly enhance CAR T cell efficacy. Th9-polarized CAR-T cells show superior efficacy in preclinical models (36). Cord blood-derived hematopoietic stem cells (HSCs) allow large-scale expansion of precursor T cells prior to terminal differentiation (33)preserving proliferative capacity and functionality during manufacturing, Together, these strategies highlight the potential of differentiation and polarization control to improve the durability of T cells therapy.

Although these strategies expand the design space for CAR-T optimization and show promise in enhancing persistence and functionality, dual costimulation may increase the risks of cytokine release and neurotoxicity (37); lineage skewing (such as Th2/Tfh bias) or unstable Th9 phenotypes limit reproducibility (38); transcriptional or epigenetic modulation introduces risks of off-target effects and autoimmunity; and large-scale production of hematopoietic stem cell–derived products remains constrained by manufacturing and regulatory hurdles. At present, control of differentiation represents the most translationally promising avenue. Future research should focus more on rational costimulatory design and the enrichment of stem cell–like T cells, in order to balance persistence with effector potency and ultimately achieve safer, more predictable, and durable clinical outcomes.

### 4.2 Aging and improvement strategies

#### 4.2.1 Improve culture conditions

Aging leads to enhanced immunosuppression in the tissue microenvironment and impairs T cell function. CAR-T cells in this environment are more likely to exhibit senescent phenotypes, thereby reducing their anti-tumor efficacy.

Optimizing the culture conditions for CAR-T cells can help mitigate this issue. IL-7 and IL-15 can direct T cells toward a memory stem cell-like phenotype, improve their expansion and viability, delay terminal differentiation, and enhance antitumor efficacy (39–41). Prolonged or high-dose IL-2 administration leads to T cell over-differentiation, whereas short-term or low-dose administration can generate early memory T cells (42, 43). Therefore, it can be seen that properly regulating the combination and concentration of cytokines is the core strategy for enhancing the function of CAR-T cells. Aging causes a decline in thymic function and T-cell generation. Supplementation with young thymic epithelial cells, gene therapy (such as the FOXN1 gene), and cytokines such as IL-7 can help restore thymic function (44).

This strategy is most immediately translatable, but dosing precision is critical. High-dose IL-2 toxicity and IL-7/IL-15 is activated abnormally in the immune system or works in synergy with other pro-inflammatory cytokines, it may be involved in the worrying pathological process of cytokine storm. Standardizing cytokine regimens across donors for GMP manufacturing is a priority.

#### 4.2.2 Extended telomere length

Telomere transfer technology slows T-cell aging by extending telomere length, thereby enhancing immune function. Telomere transfer from APC-derived vesicles to T cells, triggered by ionomycin-mediated calcium signaling, delays senescence and enhances immune activity (45–47). This strategy may play a key role in the development of therapies to enhance immune function and extend the lifespan of T cells. Artificial telomere elongation has been linked to risks of tumorigenesis and genomic instability (48). Ionomycin, although widely used as a calcium ionophore, exhibits broad cytotoxicity and poor dose controllability. In addition, the specificity and reproducibility of telomere transfer techniques remain under debate, and regulatory hurdles for clinical translation add further complexity.

#### 4.2.3 Reprogramming and gene editing techniques

T-cell reprogramming and gene editing techniques hold promise for delaying T-cell aging and enhancing antitumor immune functions. Redifferentiating T-induced pluripotent stem cells (T-IPSCs) into naïve, cytotoxic, or dedifferentiated T cells can help delay T cell depletion and aging (49–51). CLASH, a novel CRISPR system used to create CAR T cells with enhanced memory and stem cell properties by targeting genes such as PRDM1 to extend their longevity (52). I find these strategies compelling, yet their long-term efficacy and safety remain open questions. Similarly, blocking inhibitory receptors such as ILT4 shows preclinical potential to rejuvenate T cells and reprogram tumor metabolism (53), but translating these results to patients will likely face biological and regulatory hurdles.

#### 4.2.4 Regulate of signaling pathways

Regulation of signaling pathways and use of anti-aging drugs can help mitigate T cell aging. Modulating the Wnt/β-catenin and mTOR pathways has been shown to reverse T cell aging, particularly in memory T cells. And *in vivo* administration of small-molecule Wnt agonists may help achieve sustained effects (54). Drugs such as rapamycin and quercetin can restore CAR T-cell function and extend their persistence and antitumor activity by inhibiting mTOR signaling or clearing senescent cells (55). This is attractive due to oral drug accessibility, but systemic Wnt activation risks oncogenesis (56), and rapamycin causes immune suppression and metabolic toxicity (57). We advocate for short-term, perimanufacturing exposure rather than long-term systemic therapy, coupled with biomarker monitoring.

#### 4.2.5 Physical removal of senescent T-cells

Physical removal of senescent T cells can restore function and promote the expansion of memory and effector subsets. Engineered peptides can disrupt the binding of FOXO 4 and p53 to induce apoptosis in senescent cells (58). UPAR-specific CAR T cells efficiently eliminate senescent cells by targeting the surface receptor uPAR and prolonging survival in mouse models (59). These approaches highlight the potential of selectively clearing senescent T cells to enhance immunotherapy. However, concerns remain regarding off-target effects, durability of responses, and safety of translating senolytic strategies into humans, underscoring the need for careful evaluation before clinical application.

#### 4.2.6 Supplement with functional T cells

Autologous stem cell transplantation (ASCT) can restore naïve, memory, and effector T-cell function, with notable success in autoimmune diseases and hematological malignancies (60–63). Early clinical trials (NCT00133367) shows that umbilical cord blood–derived HSCs, and young stem cells more broadly, may help regenerate a youthful immune system and mitigate T-cell aging (64–66). However, such strategies face major limitations, including conditioning regimen toxicity, graft availability, risk of graft-versus-host disease, and uncertain long-term rejuvenation effects. Careful patient selection and integration with safer rejuvenation approaches will be critical for translation.

#### 4.2.7 To restore and maintain of thymic environment

Restoring and maintaining the thymic environment can reverse the effects of thymic involution, thereby promoting the production of new T-cells. Bioengineered thymic organoids incorporating cytokines such as IL-21 have been shown to enhance T-cell output in aged mice (67). Whether bioengineered thymic organoids, when combined with other cytokines or immunomodulators, can support T cell maturation and adaptability remains to be investigated. The intrathymic injection of allogeneic hematopoietic cells successfully restores functional T cell development after thymic reconstitution in a mouse model of severe combined immunodeficiency (68). However, thymic organoids still face challenges in restoring T cell function, including establishing immune tolerance, replicating complex thymic stroma, supporting thymic epithelial cells and optimizing T cell maturation (69, 70). While promising for immune rejuvenation in the elderly or immunocompromised, their clinical utility will ultimately depend on overcoming these barriers and demonstrating durable, safe T-cell reconstitution.

### 4.3 Regulation of T cell metabolism

#### 4.3.1 Glycolysis of effector cells

Effector T cells rely on glycolysis for rapid cytokine production and cytotoxicity. In the tumor microenvironment (TME), glucose competition with tumor cells impairs T cell energy supply. For instance, increased GLUT1 expression promotes glucose uptake, thereby enhancing T cell function (71). PQDN enhances the antitumor capacity of CD8+ T cells by activating the mitochondrial electron transport chain and promoting glucose uptake and glycolysis (72). While glycolytic enhancement can restore immediate function, excessive reliance accelerates exhaustion, promotes lactate accumulation, and risks metabolic toxicity. This approach is better positioned as a short-term, manufacturing-phase intervention rather than systemic therapy.

#### 4.3.2 Glycolysis and memory cells

Although glycolysis is crucial for effector T cell function, excessively high levels can hinder the long-term survival and differentiation of memory T cells (73). Restricting glycolysis using the HK2 inhibitor 2-deoxyglucose (2-DG) promoted the formation of memory CD8+ T cells and enhanced their antitumor capacity (73, 74). Metformin shifts metabolism toward fatty acid oxidation (FAO) through AMPK activation, enhancing long-term survival and antitumor capacity (75). Proper regulation of the balance between glycolysis and other metabolic pathways is crucial for optimizing CD8+ T cell function. Yet systemic drugs like 2-DG and metformin carry risks of hypoglycemia, lactic acidosis, and variable efficacy in patients with metabolic comorbidities. Their use should focus on ex vivo programming rather than chronic *in vivo* exposure.

#### 4.3.3 Glycolysis and CD8 + T cells

In TME and autoimmune settings, glycolytic suppression drives CD8^+^ T cell dysfunction (76). The combination of anti-PD-1 therapy, and glycolysis-promoting drugs can partially restore CD8+ T-cell function (77). Anti-PD-1 enhances glycolysis by inhibiting pyruvate entry into fatty acid oxidation, promoting oxidative phosphorylation (OXPHOS) and energy production, and further increasing glycolysis via the PI3K-mTOR pathway through the AGK kinase. Additionally, regulating mitochondrial function or neutralizing reactive oxygen species (ROS) can significantly improve CD8+ T-cell metabolism and enhance antitumor capacity (78). This dual strategy is compelling but risky—checkpoint blockade already predisposes to immune-related adverse events, and additional metabolic activation could exacerbate toxicity or benefit tumor metabolism. Precise timing, dosing, and biomarker-guided monitoring are critical.

#### 4.3.4 Fatty acid metabolism and oxidative phosphorylation

Quiescent T cells depend on OXPHOS, whereas activated T cells integrate glycolysis with OXPHOS (79). Upon T-cell activation, mitochondrial fragmentation reduces OXPHOS (80). AMPK activators (e.g., metformin) or mTOR inhibitors (e.g., rapamycin) stimulate FAO, promoting the generation of memory CD8+ T cells (79), thereby extending their longevity and enhancing immunological memory. Studies have shown that PGC1α and PPAR agonists (e.g., bezafibrate and fenofibrate) can enhance FAO, boosting the antitumor function of memory T cells (81–84). In adoptive cell therapy, metabolic interventions through *in vitro* reprogramming can enhance the long-term survival and anti-cancer capacity of T cells. Targeting FAO and OXPHOS holds promise for durable antitumor immunity, yet systemic administration of metabolic modulators faces significant tolerability constraints. Rapalogs and fibrates should therefore be regarded as tools for short-term, reversible metabolic programming during ex vivo expansion or transient *in vivo* windows, rather than maintenance drugs, in order to achieve a balance between long-term persistence and immediate cytotoxic activity.

### 4.4 Regulation of the tumor microenvironment

The tumor microenvironment (TME) is a complex network of tumor, stromal, and immune cells together with soluble factors that shape antitumor immunity. In this setting, T cells are suppressed by checkpoint signals (e.g., PD-1/PD-L1), regulatory and myeloid cells (Tregs, MDSCs, TAMs), and metabolic or vascular barriers, leading to immune evasion. Understanding these layers of regulation is key to developing strategies that reprogram the TME and restore T-cell activity.

#### 4.4.1 Immune checkpoint Inhibitors

PD-1 binds to PD-L1 to suppress T-cell activity, while IFN-γ and TNF-α in the tumor microenvironment increase PD-L1 expression, aiding tumor immune escape. Immune checkpoint inhibitors, including PD-1 and PD-L1 inhibitors, can block the interaction between PD-1 and PD-L1, restore T cell activity, and enable them to recognize and attack tumor cells (85). Combining immune checkpoint inhibitors with anti-inflammatory drugs or other targeted therapies, such as chemotherapy or radiotherapy, can enhance the efficacy of immunotherapy (86, 87).

The application of immune checkpoint inhibitors (ICIs) has advanced cancer immunotherapy, yet their efficacy and safety remain constrained. Some patients develop immune-related adverse events, including colitis, pneumonitis, and myocarditis. Others exhibit primary or acquired resistance, which arises from intrinsic tumor characteristics and adaptive changes within the tumor microenvironment, ultimately leading to the establishment of an immunosuppressive TME (88). Although combinations with chemotherapy, radiotherapy, or anti-inflammatory agents may enhance therapeutic efficacy, additive toxicities hinder clinical optimization. Future strategies must therefore achieve a more refined balance between efficacy and safety, rather than relying on a single immunological pathway.

#### 4.4.2 Immunosuppressive of cells

Regulation of the function of immunosuppressive cells can effectively enhance the antitumor activity of T cells, thereby improving the overall efficacy of immunotherapy.

In the tumor microenvironment, regulatory T cells (Tregs) suppress the activity of effector T cells via multiple mechanisms. Early clinical trials (NCT00888927) shows that pharmacological approaches such as PI3Kδinhibitors, anti-CTLA-4 antibodies, CCR4 antagonists, and metabolic targeting (e.g., CD36 inhibition) can reduce Treg numbers or function, thereby enhancing effector T cell responses (89–91). Other metabolic pathways such as fatty acid oxidation, glycolysis, and amino acid metabolism are also potential targets for regulating Tregs and improving the efficacy of immunotherapy.

Although these strategies may indirectly restore antitumor immune responses, systemic depletion of Tregs can disrupt immune tolerance and trigger severe autoimmune reactions (92). Therefore, future studies should prioritize the selective targeting of tumor-infiltrating Tregs and the development of delivery systems that minimize systemic toxicity without compromising self-tolerance.

Myeloid-derived suppressor cells (MDSCs) are immunosuppressive myeloid cells that proliferate widely in cancerous and chronic inflammatory environments.

Chemotherapy drugs such as gemcitabine and 5-fluorouracil have been shown to reduce the number of MDSCs (93). Targeted small-molecule drugs like SRA737 (a CHK1 inhibitor) combined with low-dose gemcitabine significantly reduce MDSC numbers, while boosting the expression of IFNβ and chemokines CCL5 and CXCL10, enhancing T cell antitumor responses (94).

MDSCs accumulate in the tumor microenvironment via chemokines, thereby hindering T cell function. Blocking CXCR1/2 (e.g., with SX-682) can reduce MDSC migration in head and neck cancer mouse models, thereby enhancing the effectiveness of NK cell immunotherapy and anti-PD-1 treatment (95–97).

Reducing the immunosuppressive activity of MDSCs can restore T-cell function and delay tumor progression. This can be achieved by inhibiting key signaling pathways such as JAK/STAT or NF-κB, or by targeting the secretion of suppressive molecules (94). PDE5 inhibitors such as sildenafil, tadalafil, and vardenafil have been shown to weaken MDSC function by reducing the secretion of inhibitory molecules (98, 99). STAT3 inhibitors, such as JSI-124 can further reduce MDSC activity (100). Reducing MDSC activity can help restore T-cell function and enhance the antitumor immune response.

Current strategies targeting MDSCs primarily involve reducing their numbers, blocking their recruitment, and inhibiting their immunosuppressive functions, thereby restoring T cell activity and enhancing antitumor immunity. However, these agents often lack specificity and may simultaneously affect normal myeloid cells, leading to bone marrow suppression and increased risk of infection (101). Moreover, the high degree of heterogeneity of MDSCs across different tumor types and patients, coupled with the absence of standardized biomarkers for their identification, has significantly limited the clinical translation of these approaches.

M2-type tumor-associated macrophages (TAMs) suppress T-cell function and promote tumor progression, making their reprogramming into M1-type macrophages an attractive therapeutic approach. Agents such as TLR agonists (3M-052, CpG ODN), anti-CD40 antibodies, and low-dose metformin can induce M1 polarization, enhance macrophage and T-cell antitumor activity, and increase T-cell infiltration into the tumor microenvironment (102–105).

Targeting TAMs with small-molecule inhibitors or nanotechnology can effectively inhibit their tumor-promoting functions and enhance the efficacy of therapies, such as immune checkpoint inhibitors.

This indicates that reprogramming TAMs not only directly enhances T cell function but also significantly improves the tumor microenvironment, offering synergistic benefits for immunotherapy. However, the inherent plasticity of tumor-associated macrophages (TAMs) often renders these effects transient, and the systemic administration of Toll-like receptor (TLR) agonists may trigger a cytokine storm (106). Recently, antibody-mediated and nanomedicine-based strategies have been explored to improve TAM targeting, yet issues such as delivery efficiency, off-target uptake, and long-term safety remain unresolved (107). Although TAM reprogramming is conceptually sound, it must be refined through targeted delivery systems and combinatorial therapies to achieve durable clinical benefits.

Future directions may also involve the integration of nanotechnology or biomaterial-based delivery systems to precisely modulate the TME, thereby enhancing therapeutic specificity and durability.

### 4.5 Regulation of depletion strategies

#### 4.5.1 PD1 maintains an exhausted state

PD1 plays a critical role in maintaining an exhausted state. By using PD1 or PD-L1 inhibitors, exhausted T cells can regain some of their functions (8), particularly their proliferative capacity and cytotoxicity. Importantly, such blockade preferentially activates progenitor-like exhausted T cells, which display superior proliferative and functional recovery compared to terminally differentiated exhausted subsets (108–110). In contrast, terminally differentiated exhausted T cells respond less to PD1 blockade but retain significant cytotoxic potential (108–110). These findings highlight that PD1 inhibition does not fully reverse exhaustion but can augment effector capacity within specific subsets. Sustained use of PD-1 blocking agents has the potential to exacerbate autoimmune responses and destabilize immune homeostasis, emphasizing the necessity of carefully balancing immune reactivation and safety considerations.

#### 4.5.2 Epigenetic regulation reverses T-cell exhaustion

Epigenetic regulation is a promising strategy for reversing T cell exhaustion, enhancing T cell function, and boosting the efficacy of immunotherapy. DNMT3A-driven *de novo* DNA methylation promotes dysfunction in CD8+ T cells, while treatment with DNA methyltransferase inhibitors such as 5-aza-2-deoxycytidine restores function when combined with PD1 blockade (111). Deleting the DNA demethylase TET2 significantly extends the lifespan of CAR-T cells and enhances their tumor-killing capacity (112). TET2 deletion prevents T cell exhaustion and promotes T cell memory formation. Early clinical trials (NCT01029366) shows that CRISPR-Cas9–mediated CD5 knockdown enhances cytotoxicity, proliferation, and survival, effects associated with increased Ras/ERK and PI3K/AKT/mTOR signaling and reduced exhaustion markers (113).

Currently, early clinical trials (NCT03179943) are evaluating the use of epigenetic drugs such as DNA methyltransferase inhibitors and histone deacetylase inhibitors (114) in combination with immune checkpoint blockers, aiming to enhance the durability of the treatment. However, the systemic nature of epigenetic therapies has raised concerns about non-targeted effects, hematotoxicity, and unexpected immune activation (115). Moreover, the optimal timing of use, dosage, and patient selection criteria have not yet been determined. Therefore, careful clinical design is still needed to maximize the benefits of epigenetic regulation and minimize long-term risks.

**Table 2** presents details on preclinical research and clinical.

**Table 2:** Problem-oriented strategy.

Optimization methods	Disease type	Research year	Mechanism	Specific benefits	Research method	Refs
MAPK inhibitors	Melanoma	2024	i. Increases MR1 expression. ii. Activate CD8+ T cells.	Enhance the activity of CD8+ T cells and promote tumor infiltration.	Experimental Research	(26)
Synthetic APC-mimetic scaffolds	NA	2020	i. Control T-cell activation. ii. Present ligands for T-cell receptors and co-stimulation.	Enhance T-cell expansion, prevent depletion, and maintain proliferation.	Experimental Research	(27)
CAR with 4-1BB and ICOS	Multiple cancer types	2018	i. Combine 4-1BB ICD with ICOS costimulation.	Improved persistence and antitumor activity of CAR T-cells.	Experimental Research	(25, 30, 31)
Regulation of Tcf1+PD-1+ T cells	Melanoma	2019	i. Propertie to sustain immune response.	Improved immune response, tumor control, and T-cell persistence in immunotherap.	Experimental Research	(25, 30, 31)
Modulation of NR4A expression	Infections and cancer	2021	i. Regulate T cell differentiation, activation, and persistence by controlling early activation and memory T cell differentiation.	Improved T cell activation, enhanced differentiation into memory T cells, and better immune responses in infections and cancer.	Review	(32)
Th9 polarization techniques	Leukemia and Liver cancer	2020	i. IL-4, TGF-β, anti-IFN-γ antibody, and IL-2 polarize human T cells into Th9 subtype.	Enhance antitumor activity, strengthen immune response.	Experimental Research	(36)
Cord blood stem cells	NA	2023	i. Avoids the need to suppress the endogenous T cell receptor. ii. Avoids terminal differentiation.	Prevent terminal differentiation, maintain T cell functionality.	Review	(33)
IL-7 and IL-15	Leukemia	2013	i. Drive the generation and expansion of TSCM.	The strategy enhances the persistence and self-renewal of TSCM cells and improves the longevity and efficacy of T cells in patients, particularly in those with acute leukemia.	Experimental Research	(39–41)
Short-term/low-dose IL-2	Leukemia	2017	i. Drive the activation and expansion of T cells. ii. Promote memory T-cell differentiation. iii. Minimize the negative effects of high-dose IL-2, such as immune suppression.	Generate early memory T cells, prolong persistence.	Experimental Research	(42, 43)
Telomere transfer technology	NA	2022	i. Prevent T-cell senescence. ii. Enhances their ability to generate long-term immunological memory.	Prevent T cell aging, extend functional lifespan.	Experimental Research	(45–47)
CLASH CRISPR system	Cancer	2024	i. Harness the Cas12a/Cpf1 system for gene editing. ii. Edit PRDM1 in CAR-T cells.	This system promotes increased T-cell proliferation, stem-like properties, central memory, and T-cell longevity.	Review	(52)
Blocking ILT4	Lung cancer, melanoma, prostate cancer and breast cancer	2020	i. Prevent the interaction between ILT4 receptors and their ligands.	Reduce T cell aging, enhance antitumor immune response.	Experimental Research	(53)
Activating Wnt/β-catenin pathway	Melanoma, lung cancer and prostate cancer	2009	i. Regulate genes involved in memory T-cell formation. ii. Stabilize β-catenin levels.	Maintain long-term T cell memory, improve survival.	Experimental Research	(54)
Inhibiting mTOR pathway	NA	2014	i. Reprogram cellular metabolism. ii. Allow T cells to function better even in elderly individuals.	The inhibition of the mTOR pathway was evaluated by measuring the immune response to influenza vaccination, with a particular focus on T cell activation and proliferation.	Experimental Research	(55)
ASCT	Autoimmune diseases	2016	i. Renew the Treg pool. ii. Modulate T effector cells. iii. Improve immune tolerance.	Restore immune function, enhance antitumor activity.	Experimental Research	(60–63)
Cord blood stem cells	Immune deficiency or autoimmune conditions	2014	i. Promote the generation of naive T cells in the thymus. ii. Enhance T cell diversity and function.	Generate young, functional T cells, prevent premature aging.	Clinical trial(NCT00133367)	(64–66)
Boosting GLUT1 expression	NA	2019	i. Increase glucose uptake to support T cell metabolism and activation.	Increase energy uptake in T cells, boost antitumor capacity.	Review	(71)
Using PQDN to activate the mitochondrial electron transport chain	Low immunogenic tumors	2022	i. Augment TCR-mediated recognition of tumor antigens.	Provide rapid energy, enhance anti-infection and antitumor capacity.	Experimental Research	(72)
Activating AMPK	Viral Infections	2018	i. Promote memory T cell differentiation.	Modulate the balance of effector vs. memory precursor effector cells.	Experimental Research	(73–75)
Reprogramming mitochondria	Colon carcinoma and fibrosarcoma	2018	i. Enhance mitochondrial function, OXPHOS, and FAO to support CD8+ T cell function in tumors.	Improve cellular metabolic efficiency and optimize immune response.	Experimental Research	(81–84)
PD-1/PD-L1 inhibitors	Osteoarthritis	2022	i. Activate JAK/STAT signaling and transcription factors. ii. Alter chromatin structure and PD-1 gene transcription.	Restore function of T cells, improves T cell activation or suppresses exhaustion depending on the cytokine milieu in TME.	Review	(85)
Combination with anti-inflammatory drugs	Melanoma, Lung Cancer and other solid tumors	2017	i. Enhance the efficacy of anti-PD-1 therapy.	Enhance TIL survival. Improve T cell infiltration and anti-tumor immunity. Reduce PD-L1 and TIM-3 expression in tumor microenvironment.	Experimental Research	(86, 87)
Targeting Tregs	Cutaneous T-cell lymphoma	2015	i. Target CCR4 on Tregs and malignant T cells, reducing Treg numbers.	Reduce Treg-mediated suppression.	Clinical trial(NCT00888927)	(89–91)
Inhibiting the migration of MDSCs	Pediatric rhabdomyosarcoma	2020	i. Inhibite CXCR2 to reduce MDSC migration.	Decrease immune suppression, restore T cell function and enhance anti-PD1 response.	Experimental Research	(93–100)
PDE5	Colon, breast, melanoma and HCC	2006	i. PDE5 inhibitors target MDSC activity by inhibiting their immunosuppressive molecules.	Increase T-cell infiltration, activation and proliferation. Reduce tumor growth.	Experimental Research	(93–100)
Reprogramming M2-TAMs to M1 phenotype	Pancreatic cancer and other solid tumors	2020	i. CD40 agonists activate macrophages. ii. The upregulation of CCL5 promotes the infiltration of CD4+ T cells into the tumor.	Enhance T cell-mediated anti-tumor immunity. Increase tumor infiltration by CD4+ T cells.	Experimental Research	(102, 103)
Inhibiting DNMT3A	Colorectal cancer, ovarian cancer and melanoma	2021	i. Reverse T cell exhaustion and restore T cell function. ii. Remove the state of DNA hypermethylation.	Reverse T cell exhaustion, increase cytokine production, improve T cell survival, and enhance anti-tumor immunity.	Experimental Research	(111)
TET2 gene knockout	CLL	2021	i. Promote an epigenetic reprogramming that favors a central memory T-cell phenotype.	Improve CAR T-cell proliferation, memory phenotype, persistence, and enhance tumor-killing capability.	Clinical trial	(112)
CD5 knockout	ALL and other solid tumors	2024	i. Increase the activation of signaling pathways critical for T cell function(e.g., Ras/ERK and PI3K/AKT/mTOR) while reducing T cell exhaustion markers.	Enhance T cell proliferation and survival. Reduce exhaustion.	Experimental Research and clinical trial(NCT01029366)	(113)

### 4.6 Aging, metabolic dysregulation, and TME-mediated immunosuppression: interconnected barriers to T Cell Function

T cell function in cancer is limited by a convergence of aging, metabolic dysregulation, and tumor microenvironment (TME)-mediated immunosuppression. Aging is associated with immunosenescence, marked by diminished T cell proliferation, reduced memory formation, and impaired metabolic flexibility. Upon activation, T cells undergo profound metabolic remodeling: effector T cells rely on aerobic glycolysis to fuel proliferation and cytotoxicity, while memory T cells depend on fatty acid oxidation (FAO) and oxidative phosphorylation for long-term persistence. Dysregulation of these metabolic pathways—exacerbated by tumor-driven nutrient depletion, lactate accumulation, and inhibitory signaling (e.g., PD-1/PD-L1)—compromises T-cell survival and effector function. Simultaneously, the TME reinforces suppression through physical and cellular barriers: dense stromal fibrosis limits infiltration in pancreatic and ovarian cancers, CAF-derived CXCL12 repels T cells in melanoma, and MDSCs in colorectal and lung cancers release arginase and reactive oxygen species that blunt T-cell activity. VEGF and abnormal vasculature further hinder T-cell trafficking.

Together, these age-related, metabolic, and TME-derived factors synergize to constrain antitumor immunity and diminish the efficacy of immunotherapy. Therefore, rational combinatorial strategies hold strong promise—for example, pairing metabolic interventions (enhancing FAO in memory T cells, inhibiting tumor glycolysis) with TME-targeting therapies (anti-VEGF treatment, CAF inhibition), while considering the impact of immunosenescence. Such approaches may restore T-cell resilience and maximize the clinical benefit of cancer immunotherapy.

## 5 Conclusions

Recent breakthroughs in pharmacologically enhancing T cell function—including small-molecule agonists, checkpoint-blocking biologics, and genetically engineered cellular therapies—have redefined the landscape of tumor immunopharmacology. Despite these advances, critical hurdles persist, such as T cell metabolic collapse in hypoxic and nutrient-deprived tumor niches, antigen-driven terminal exhaustion, and immune-related adverse events linked to systemic immune activation. For solid tumors, the effects of T cell therapy remain limited because of the complexity of the tumor microenvironment. Emerging strategies—ranging from precise genetic editing and metabolic reprogramming to targeting novel immunoregulatory pathways and engineering next-generation T cells—are now providing concrete opportunities to overcome these barriers. Importantly, the field must prioritize translating these innovations into clinically actionable frameworks by systematically testing combination regimens, tailoring interventions to distinct immune phenotypes, and implementing real-time monitoring of T cell dynamics in patients. Future progress will also depend on integrating AI-driven drug discovery, single-cell multi-omics–based patient stratification, and adaptive clinical trial designs. To accelerate this trajectory, stronger collaboration across immunology, bioengineering, computational biology, and clinical oncology will be essential, ultimately enabling the development of T cell therapeutics that are both context-specific and broadly applicable across diverse tumor immune landscapes.
